# Characterization of the natural enemy community attacking cotton aphid in the Bt cotton ecosystem in Northern China

**DOI:** 10.1038/srep24273

**Published:** 2016-04-14

**Authors:** Abid Ali, Nicolas Desneux, Yanhui Lu, Bing Liu, Kongming Wu

**Affiliations:** 1 State Key Laboratory for Biology of Plant Diseases and Insect Pests, Institute of Plant Protection, Chinese Academy of Agricultural Sciences, Beijing 100193, P. R. China; 2 Department of Entomology, University of Agriculture, Faisalabad 38000, Pakistan; 3 INRA (French National Institute for Agricultural Research), Univ. Nice Sophia Antipolis, CNRS, UMR 1355-7254, Institut Sophia Agrobiotech, 06903, Sophia Antipolis, France; 4 College of Horticulture and Plant Protection, Yangzhou University, Yangzhou, 225009, China

## Abstract

Planting Bt cotton in China since 1997 has led to important changes in the natural enemy communities occurring in cotton, however their specific effect on suppressing the cotton aphids (being notorious in conventional cotton ecosystem) has not been fully documented yet. We observed strong evidence for top-down control of the aphid population, e.g. the control efficiency of natural enemies on cotton aphid increased significantly in open field cages compared to exclusion cages, accounted for 60.2, 87.2 and 76.7% in 2011, 2012 and 2013 season, respectively. The cotton aphid populations peaked in early June to late July (early and middle growth stages) in open field cotton survey from 2011 to 2013. The population densities of cotton aphids and natural enemies were highest on middle growth stage while lowest densities were recorded on late stage for aphids and on early plant stage for natural enemies. Aphid parasitoids (*Trioxys* spp., *Aphidius gifuensis*), coccinellids and spiders were key natural enemies of cotton aphid. Briefly, natural enemies can suppress aphid population increase from early to middle plant growth stages by providing biocontrol services in Chinese Bt cotton.

Cotton, *Gossypium hirsutum* L. (Malvaceae), is an important cash crop that plays a vital role in the agriculture sector of the Chinese economy. However, cotton crops are infested by various insect pests (i.e. about 30 species are common) throughout the growing season in China^1^. In the conventional Chinese cotton planting ecosystem before 1997, cotton bollworm (CBW, *Helicoverpa armigera* H.) was of great economic importance and caused significant yield reduction. To manage CBW in cotton, the Chinese government approved the commercial use of transgenic Bt cotton in 1997^2^. The conventional cotton ecosystem shifted to the Bt cotton ecosystem in part due to high pest pressure of CBW and decreasing effectiveness of pesticides. Successful control of CBW through adoption of Bt cotton led to a steadily increase in Bt cotton planting in Eastern and Northern China. The widespread adoption of Bt cotton in Northern China had effectively reduced the use of insecticides and promoted the biocontrol services by natural enemies^3^.

Cotton aphid (*Aphis gossypii* L.) has been long considered as an important secondary pest in Bt cotton fields^1^. The high density of cotton aphid due to the insecticide resistance^4^ and/or favorable weather condition has a negative impact on the yield^1^,^5^,^6^. Natural enemies are of great importance in suppressing insect pests populations in agricultural systems^3^,^7^,^8^,^9^,^10^. As far as the Chinese Bt cotton ecosystem is concerned, one year field cage study was performed to measure the top down forces on aphid population growth in central China^11^. In the Yellow River Region (YRR), the impact of natural enemies to reduce the population density of cotton aphid was reported^12^. However, there has been no record of the seasonal population dynamics of cotton aphid and their natural enemy species among different plant stages throughout the growing season. Therefore, the work reported herein was conducted (i) to assess the specific effects of natural enemies on cotton aphid population dynamics using various natural enemy exclusion cages experiments and artificially released aphid populations, (ii) to monitor the population dynamics of aphid and associated natural enemy species at different growth stages (early, middle, and late) by open cotton field survey, and (iii) to identify the most abundant natural enemy species (especially parasitoids) of cotton aphid in Bt cotton fields of Northern China. The results of the present study will help to characterize changes in the natural enemy community in the current Bt cotton agro-ecosystem along the YRR of China.

## Results

### Field cage experiment

**Figure 1:**
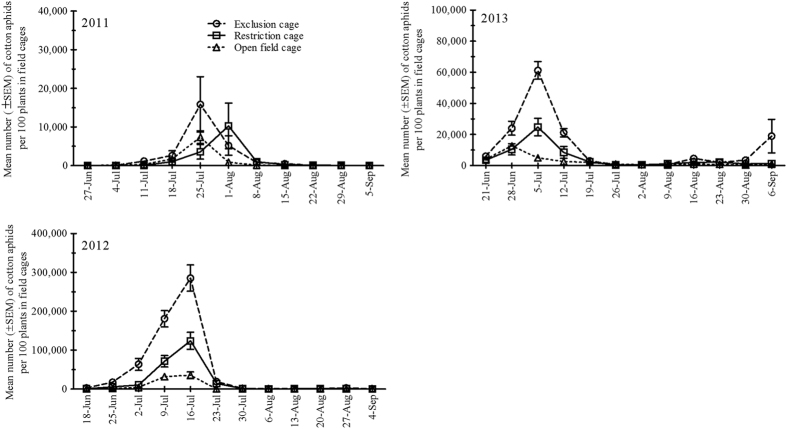
Mean population dynamics (± SEM) of cotton aphids per 100 plants surveyed in the cotton field of various natural enemy cages treatments (restriction, exclusion and open field cages) from Mid-June to early September for three growing seasons; 2011, 2012 and 2013 in Langfang experimental station (Yellow River Region of China).

In 2011, cotton aphid densities did not differ significantly among blocks (*P* = 0.299) and cage types (*P* = 0.552). But as function of the dates (*P* < 0.001), and the two factors (cage type and sampling date) interact significantly (*P* < 0.001) (Table 1). The lowest mean number of aphids (944 per 100 plants) was recorded in the open field cages, whereas the highest mean number of aphids (2,370 per 100 plants) was recorded in exclusion cages (Fig. 1). In 2012, cotton aphid densities recorded did not differ significantly among blocks (*P* = 0.719), but aphid densities differed significantly among cage types (*P* < 0.001), as function of the dates (*P* < 0.001), and the two factors (cage type and sampling date) also interacted significantly (*P* < 0.001) (Table 1). In 2012, the lowest mean number of aphids (6,090 per 100 plants) was recorded in the open field cages, whereas the highest mean number of aphids (47,918 per 100 plants) was recorded in exclusion cages (Fig. 1). In 2013, cotton aphid densities did not differ significantly among blocks (*P* = 0.062), but aphid densities differed significantly among cage types (*P* < 0.001), as function of the dates (*P* < 0.001), and the two factors (cage type and sampling date) also interacted significantly (*P* < 0.001) (Table 1). In 2013 season, the lowest mean number of aphids (2,574 per 100 plants) was recorded in the open field cages, whereas the highest mean number of aphids (11,731 per 100 plants) was recorded in exclusion cages (Fig. 1). Briefly, in 2011 and 2013, cotton aphid numbers were lower than 2012.

### Open field survey

In the open cotton field survey, the lowest (1,399 per 100 plants) and the highest (26,346 per 100 plants) mean number of aphids was recorded in 2011 and 2012 season, respectively. The most common groups of natural enemies recorded in the natural enemy guild were coccinellids (2011), aphid parasitoid (2012) and spiders (2013) (Table 2). *Aphidius gifuensis* A. was the most abundant aphid parasitoid during all seasons.

#### Population dynamics of cotton aphid

**Figure 2:**
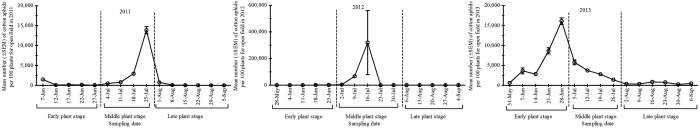
Mean population dynamics (± SEM) of cotton aphids per 100 plants surveyed during three cotton growth stages {Early plant stage (May-June), Middle plant stage (July) and Late plant stage (August- September)} from end of May to early September in the open cotton field survey for three growing seasons; 2011, 2012 and 2013 in Langfang experimental station (Yellow River Region of China).

Overall, there were substantial differences in aphid population density over the sampling period during the three seasons. The aphid population density peaked in July 23^rd^, July 16^th^ and June 25^th^ during summer in 2011, 2012 and 2013, respectively (Fig. 2). Furthermore, the highest population density of cotton aphid was recorded at middle plant stage in both 2011 and 2012 seasons whereas it occurred at early plant stage in 2013 (supplementary data - Fig. S3a).

#### Population dynamics of natural enemies

**Figure 3:**
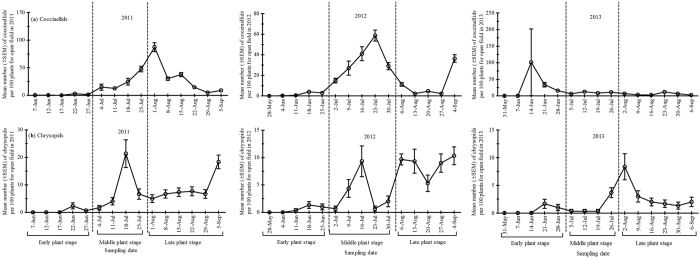
Mean population dynamics (± SEM) of (**a**) coccinellids and (**b**) chrysopids per 100 plants surveyed during three cotton growth stages {Early plant stage (May-June), Middle plant stage (July) and Late plant stage (August- September)} from end of May to early September in the open cotton field survey for three growing seasons; 2011, 2012 and 2013 in Langfang experimental station (Yellow River Region of China).

**Figure 4:**
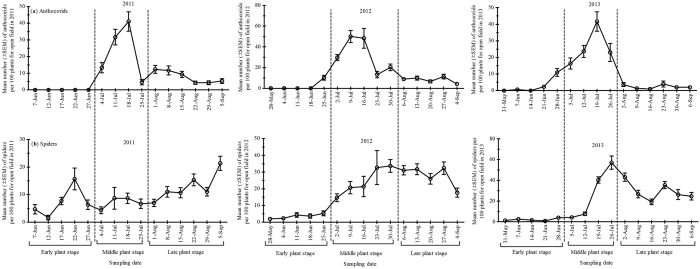
Mean population dynamics (± SEM) of (**a**) anthocorids and (**b**) spiders per 100 plants surveyed during three cotton growth stages {Early plant stage (May-June), Middle plant stage (July) and Late plant stage (August- September)} from end of May to early September in the open cotton field survey for three growing seasons; 2011, 2012 and 2013 in Langfang experimental station (Yellow River Region of China).

**Figure 5:**
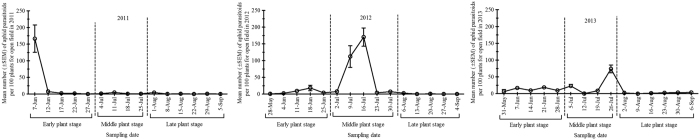
Mean population dynamics (± SEM) of aphid parasitoids per 100 plants surveyed during three cotton growth stages {Early plant stage (May-June), Middle plant stage (July) and Late plant stage (August- September)} from end of May to early September in the open cotton field survey for three growing seasons; 2011, 2012 and 2013 in Langfang experimental station (Yellow River Region of China).

The most abundant natural enemy group differed according to the seasons and plant growth stages considered. Among predators complex, coccinellids and spiders were the most common species, followed by anthocorids and chrysopids from 2011–2013 (Table 2). Overall, natural enemy distribution at early, middle and late plant growth stages was recorded as 26, 38 and 36% in 2011 while 6, 70 and 24% in 2012 whereas 26, 50 and 24% in 2013, respectively (Table 3). Coccinellid populations peaked in August 1^st^, July 23^rd^ and June 14^th^ during summer 2011, 2012 and 2013, respectively (Fig. 3a). Furthermore, the highest population density of coccinellids was recorded at late plant stage in 2011, middle plant stage in 2012, and early plant stage in 2013 (supplementary data - Fig. S3b). Population density of chrysopids peaked in July 18^th^, July 16^th^ and August 19^th^ during summer 2011, 2012 and 2013, respectively (Fig. 3b). In addition, the highest density of chrysopids was recorded equally at middle and late plant stages in 2011, and late plant stage in both 2012 and 2013 (supplementary data - Fig. S3c). Overall, anthocorids ranked third most common predators in cotton field after the coccinellids and spiders. The anthocorid populations peaked in July 18^th^, July 9^th^ and July 19^th^ during summer 2011, 2012 and 2013, respectively (Fig. 4a) and the highest density was always recorded at middle plant stages during 2011-2013 (supplementary data - Fig. S3d). Spiders ranked second most common predator in cotton fields studied. Spider populations peaked on June 22^nd^, July 30^th^ and July 26^th^ during summer 2011, 2012 and 2013, respectively (Fig. 4b). The highest spider density was recorded at late plant stage during 2011-2013 (supplementary data - Fig. S3e). The density of aphid parasitoid mummies varied during the sampling period during three seasons. Overall, density of aphid parasitoids ranked third most common natural enemy after coccinellids and spiders (Table 2). Population density of aphid parasitoids peaked in June 7^th^, July 16^th^ and July 26^th^ during summer 2011, 2012 and 2013, respectively (Fig. 5), with the highest density recorded at early plant stage in 2011, and at middle plant stage in both 2012 and 2013 (supplementary data - Fig. S3f).

Aphid parasitoids were largely attacking cotton aphid during 2011 when there was high population density of aphids at early seedlings stage. In 2012 and 2013, aphid parasitoids played a combined role with predators for aphid reduction throughout the season. Aphidiines were primarily observed as only 15 Aphelinidae mummies were seen during the whole study. Randomly collected mummies (stored in growth chamber) in 2012 and 2013 yielded parasitoids and hyperparasitoids. In early cotton stage, in 2012, 52% of collected mummies yielded hyperparasitoids (total collected = 69) while only hyperparasitoids were recorded in 2013 for that period. In middle cotton stage, in 2012, 46% of collected mummies yielded hyperparasitoids (total collected = 108) while 6% hyperparasitoids were recorded in 2013 for that period. In late cotton stage, in 2012, none of collected mummies yielded hyperparasitoids while 100% hyperparasitoids (total collected 34) were recorded in 2013 for that period. Numbers of primary parasitoids and hyperparasitoids at early and middle growth stages were higher than at the other stage during both seasons of the cotton crop, respectively. At early growth stage, in 2012, primary parasitoids emerged were *A. gifuensis* (97%) and *Trioxys* ( = *Binodoxys*) spp. (3%). By contrast, in 2013, only *Trioxys* spp. was observed (100%). At middle growth stage, in 2012, primary parasitoids were *Trioxys* spp. (91%) and *A. gifuensis* (9%) whereas in 2013, only *Trioxys* spp. was observed (100%). At late growth stage of the cotton crop, 100% *Trioxys* spp. were recorded only in 2013.

## Discussion

The differences in the population density of cotton aphid recorded in different exclusion cages showed a strong top-down impact of natural enemies over three growing seasons from 2011–2013. The numbers of aphid remained low from middle June to early July (early to middle plant growth stage of the crop). In open cotton field survey, population dynamics of aphids varied with the seasons and stages of plant growth where the highest population density of cotton aphid was recorded during 2012, and at the middle plant growth stage (July) over three growing season. More specifically, early and middle growth stages of cotton crop are the most critical for aphid infestation as a sudden increase in the density could be observed. Among the natural enemy guild, the most common species recorded were coccinellids, spiders, and aphidiine parasitoids in three sampling seasons where the highest numbers of natural enemy was recorded at middle plant growth stages of the cotton crop (see Table 2). Potential aphid parasitoids were identified as *Trioxys* spp. and *Aphidius gifuensis* A. during two sampling season (2012 and 2013). Overall, these results suggest that natural enemy populations should be conserved by avoiding insecticide application at early plant growth stage of cotton crop that would ultimately suppress the increasing numbers of cotton aphid populations at middle and late planting growth stages. Moreover, this series of experiments is very useful information to highlight and provide a direct assessment of the seasonal importance of different natural enemy groups attacking cotton aphid in the Bt cotton ecosystem along YRR of Northern China.

Exclusion cages experiments evaluated a great contribution of natural enemies against cotton aphid density over three growing seasons in transgenic Bt (Cry1Ac) cotton field in Northern China. Similar findings have been reported by Lin *et al.*^12^ and Han *et al.*^11^ in Bt cotton ecosystem of Northern and central China, respectively^12^,^11^. When there were no natural enemies (i.e. in exclusion cages), cotton aphid populations could increase up to maximum of 317-fold in 2011, 5703-fold in 2012 and 1223-fold in 2013 (from the aphid density at the initial release date). Several studies on population dynamics of cotton aphid and its natural enemies in cotton field without any insecticide use had been conducted in Northern China, and the results showed that natural enemies could not effectively suppress the aphid population^13^,^14^. However, in the open field in this 3-year study from 2011-2013, cotton aphid populations were largely reduced regardless of initial infestation levels. It indicated the control efficiency of natural enemies on cotton aphid had increased in cotton field after wide-scale adoption of Bt cotton^3^. A distinct but additive effect on the population density of cotton aphid was observed when natural enemies (both predators and parasitoids) had access to the aphids as in case of open field cages, and cotton aphid population reached a maximum of 147-fold in 2011, 707-fold in 2012 and 253-fold in 2013. The cage effect was not significant only during 2011 season (results summarized in Fig. 1; Table 1), which might result from the low seasonal mean population density of aphids recorded during 2011, which was 7,500 aphids per 100 plants, 16.6 and 4.3 times less than aphid density recorded in 2012 and 2013, respectively. Safarzoda *et al.*^15^ reported non-significant effect of exclusion cages during the low aphid density season^15^. These major differences in cotton aphid population dynamics indicated strong, but not systematic, top-down influence of natural enemies on cotton aphid in fields.

In our visual observations when predators were present in the open field cages, the parasitoid population density remained low during the whole season. Two possibilities could exist for this trend (i) either because of possible intra-guild predation (IGP) of parasitoid mummies by coccinellids^16^,^17^,^18^,^19^,^20^, and/or (ii) through resource competition of parasitoids with the generalist predators in cages {for more details, see Fig. 1 (2012 aphid population density)}^21^,^22^. In this case, the aphid parasitoids may help reducing aphid densities but primarily in mid-season (each season in July) as population dynamics change between years and is affected by many factors. The results of cages showed the impact of natural enemies by fluctuation in cotton aphid population using exclusion cages.

The open field survey confirmed the prevalence of parasitoids, coccinellids and spiders on the cotton aphid in Bt cotton field. Visual presence of *C. septempunctata*, *H. axyridis*, *P. japonica* and *A. variegata* matches with other studies which highlighted the role of *C. septempunctata* as major predator responsible for variation in population of cotton aphid in northern China^23^,^24^. *P. japonica* is reported as one of the most common predators of cotton aphid because of its life history phenology and features that proved its importance as useful biocontrol agent for the management of the aphid in cotton fields^25^,^26^. Coccinellids are important natural enemies of several aphid species^27^,^28^, while Lu *et al.*^3^ reported the presence of the same coccinellid species playing an important role in the suppression of cotton aphids in cotton fields of Northern China^3^. More specifically, coccinellids as general predators can feed on various other sucking insects like thrips, spider mites, whiteflies and many other small prey^29^,^30^,^31^,^32^, these alternate hosts were also present during our study at middle to late growth stages of the crop but were not monitored. Harwood *et al.*^31^ found that these prey can help the predators to establish in the early season when aphid density is low^31^. In short, coccinellids have great effect to reduce or delay the establishment of aphids and thereafter their subsequent population density in the early season of seedling stage^33^. In this way generalist predators are very useful as biocontrol agent for conservation biological control. Among Araneae (spiders), members from Linyphiidae and Thomisidae were visually observed attacking cotton aphid in Bt cotton fields of Northern China. Sheet-web weavers, *Erigonidium graminicolum* S., and Hunting spiders, *Misumenopos tricuspidata* F. and *Pardosa t-insignita* Boes. et Str. (Lycosidae) were reported as the most common species of spiders in Northern China^34^. For centuries, spiders have been used in Chinese field crops as a tool for management of rice pests^35^. Spiders were reported as good generalist predators due to their obligate predatory feeding strategies^36^,^37^,^38^,^39^. Spiders have potential to cause mortality of crop pests such as aphids^40^. A complex of spider species is more effective at controlling prey densities (including aphids) than the presence of a single species of spider^41^. In our study we found a complex of two above mentioned spider’s families. Spiders do have the potential to be highly effective biological control agents as stressed in our study, notably during the last season (2013) where spiders were the most abundant natural enemies to suppress aphid population. However further studies including gut content analysis, would confirm it^42^. Parasitoids, *Trioxys* ( = *Binodoxys*) spp. and *A. gifuensis* proved to be major natural enemies (species identified from randomly collected aphid parasitoids) for suppressing cotton aphid populations density in Bt cotton fields of YRR of China. However, Aphidiinae alone could not be factor to limit totally aphid population build up as aphid density reached ~123,000 aphids per 100 plants by July 16^th^ in 2012 season. There might be a rapid aphid population growth in these restriction cages at early cotton growing season because predators were excluded (as general predators are known to prevent pest population build up early in the season)^43^.

Among the aphid parasitoids, *Aphidius* spp., are being successfully used in wide range of crops across the world^44^. In the past, various studies were conducted to report the diversity of natural enemy species in cotton in different regions of the China (e.g. in cotton fields along YRR of China near Beijing), and the dominance of *Chrysoperla sinica* T., *P. japonica*, various spiders and *Orius minutus* L. was reported^29^. In our findings, aphid parasitoids (*A. gifuensis*) were the most abundant species during the 2012 season, while coccinellids were the most abundant in 2011 but Men *et al.*^45^ reported the decrease in diversity of natural enemies during three year studies (1999, 2000 and 2001) in Bt-cotton of Northern China^45^. In another study, *A. gifuensis, P. japonica* and *C. septempuctata* were recorded dominant in cotton^46^. There were similar findings in Hebei province of Northern China where the dominance of *P. japonica* was reported^47^. We found that aphelinid parasitoids were almost absent from the field (as reported in *Brassicae* crops)^48^,^49^. *Trioxys* spp. and *A. gifuensis* were the most common aphid parasitoids over two growing seasons (2012 and 2013). Several species from the *Binodoxys* genus ( = *Trioxys*) are known to efficiently attack cotton aphid^50^,^51^, and *B. indicus* or *Trioxys indicus* may be important natural enemies of this aphid pest in the YRR region and other cotton growing regions that have not yet been extensively surveyed.

Overall, the abundance of natural enemies especially the predators and aphid parasitoids, both in early and middle growth stages of the cotton crop presents a challenge to insect pests management researchers to develop sustainable biological control conservation techniques. If succesful in developping such optimized IPM, then it would help to manage outbreaks in populations of secondary pests in Bt cotton ecosystem along YRR in Northern China.

## Methods

### Field area and aphid colony

Experiments were conducted during summer each year from the end of May to early September in 2011, 2012 and 2013 at Langfang experimental station, Institute of Plant Protection (IPP), Chinese Academy of Agricultural Sciences (CAAS), Hebei Province of Yellow River Region of China (116.4˚ E, 39.3˚ N). The genetically modified cultivar “Zhong zhi mian” which produces Cry1Ac protein was used during the experiments. The seeds were provided by Langfang experimental station, China. The experimental field was divided into two parts: field cage experiments and open field survey. For the field cage experiment, cotton was planted in 13 m × 52 m (0.167 acre), and for the open field survey cotton was planted in 16 m × 52 m (0.21 acre). Cotton was planted on May 5^th^, 12^th^, and 14^th^ in 2011, 2012, and 2013, respectively. The cotton was harvested in October in all growing seasons and then the field was plowed, fertilized, and irrigated before the sowing of cotton for the next year. Cotton seed was mechanically sown 5 cm deep at 20 kg/30,000 plants per ha at a plant, row and bed spacing of 40, 40 and 100 cm, respectively in all seasons. Cotton seedlings emerged 8–10 days after planting in all growing seasons. All agronomic practices of the cotton were followed according to local recommendations in which the experimental station is located. No pesticides were applied to the field.

To provide aphids for artificial infestation in exclusion cages experiments, naturally occurring cotton aphids were collected from this field in May for all the seasons. Aphids were cultured on the same cotton variety of 3–10 days old seedlings in plastic pots in a greenhouse (at 25 ± 1 °C, 60–70% RH and a photoperiod of 16: 8 (L:D) hour) at Langfang experimental station.

### Experimental setup

#### Field cage experiment

Three levels of natural enemy exclusion cages were used: (i) exclusion cage with 1 × 1-mm mesh openings in which there was no entry of any predator or parasitoids and thus the aphids were fully protected, (ii) restriction cage with 2 × 2-mm mesh openings in which the activities of predators were restricted but allowed aphid parasitoids to enter the cages, (iii) open field cages with four bamboo wood sticks without using any mesh standing upright into the ground. This treatment allowed natural enemies complete access to the aphids. Similar cage type and mesh size were used before in cotton fields^12^.

Three different treatments were established on June27^th^ in 2011, June 18^th^ in 2012 and June 21^st^ in 2013 where all treatments were replicated six times with 60 healthy plants per replicate for each of three blocks following the completely randomized block design (see supplementary data, Fig. S1). Three blocks, each of 13 m × 15 m with 2 m buffer surrounding the blocks. Each cage/plot (1.8 × 2 × 2 m, length × width × height) was set over three rows of two planting beds. Ten healthy plants inside each cage/plot were selected and marked with plastic strips.

Exclusion and restriction cages were of polyester sacks 2 m in width, 1.8 m in length and by 2 m in height and supported on iron poles at each corner. There was 1.0 m and 0.80 m distance between cages (Fig. S1). The bottom edges of the mesh were buried in the soil up to a depth of 10 cm to prevent or exclude the ground-dwelling predators.

One day before artificially infesting the plants with aphids, the selected plants were cleaned for any resident arthropods manually by camel’s hair brush. To infest plants, the aphids were placed on the highest central leaflet to the experimental plants by using a small and fine camel’s-hair brush. In each replicate, the plants were infested artificially at the rate of 5 aphids per plant (adults) at June 20^th^, 11^th^ and 14^th^ in 2011, 2012, and 2013 season, respectively. After the aphid infestation on plants, cages were closed by a zipper opening on one side, and aphids inside each treatment were left to reproduce for seven days. Samples of aphid density on each plant (as a whole) were visually examined and counted by weekly survey each year from Mid-June to early September.

### Open field survey

The field was divided into three blocks every season and each block was 16 m × 15 m with 2 m buffer surrounding the blocks. Twenty plots were selected as fixed sampling sites in each block following the five plants method used in soybean field^52^. Each plot consisted of five plants and at least one of these plants had been naturally colonized by the aphids (see supplementary data, Fig. S2). Twenty plots were established on June 7^th^ in 2011, May 28^th^ in 2012 and May 31^st^ in 2013. Each plot was 1.8 m × 2 m while plot to plot distance was 1 m during all seasons. In open field survey, sampling was started on June 7^th^ in 2011 at five days interval for first four times, and in 2012 and 2013, started on May 28^th^ at 7 days interval throughout the all seasons (at this stage cotton was at 4–6 leaves stage).

Sampling during the open field survey was carried out as follow: Each plant (as a whole) was visually examined and counted for all stages (larvae, nymphs, adults) of the following arthropods; cotton aphid, coccinellids, chrysopids, anthocorids, spiders and aphid parasitoids. All the predators were identified to order. Samples were collected during three cotton growth stages; (i) early plant stage (May-June) at seedling and square formation, (ii) middle plant stage (July) at flowering and boll formation, and (iii) late plant stage (August- September) at boll formation and opening, and before harvesting. Aphid parasitoids were counted on the basis of their field appearance as tan (Aphidiinae) and black (Aphelinidae). Mummy samples were collected randomly in 2012 and 2013 from various open field plots (when parasitoid densities were at high levels) for further identification of parasitoids. The collected mummies (2012: n = 177, 2013: n = 150) were brought back to the laboratory and placed individually in gel caps in a climatic chamber (25 °C, 65% RH and 16:8 h/ L:D) for 10 days. The emerged parasitoids were identified using identification keys^53^,^54^,^55^,^56^,^57^. Data sheets are stored at IPP-CAAS, Beijing, P. R. China.

### Statistical analyses

Aphid densities in the field cage experiment were non-normally distributed and therefore were log transformed for analyses. Counts were converted into mean number ( ± SEM) per 100 plants in both experiments: 10 plots/cages for exclusion field cage experiments, and 20 plots in open field survey. For exclusion cage experiments, we tested the effects of block, cage type, sampling date, and interaction between sampling date and cages level on aphid density using PROC MIXED repeated measures ANOVA with SAS program, version 9.2^58^. Sample date was repeated within replicates and separate analyses were carried out for each year of the study. A probability level of *P* < 0.05 was considered as indicating statistical significance separately for each year of the study. For survey data, comparison of three cotton growth stages (early, middle and late stage) for all sampling parameters (cotton aphid and natural enemies) during three sampling years (2011, 2012 and 2013) were carried out using a One-way analysis of variance with the Student-Neuman-Keuls test (SAS program, version 9.2)^58^. A probability level of *P* < 0.05 was considered as indicating statistical significance separately inside each year of the study. GraphPad Prism version 6.00 was used for drawing all the graphs.

## Additional Information

**How to cite this article**: Ali, A. *et al.* Characterization of the natural enemy community attacking cotton aphid in the Bt cotton ecosystem in Northern China. *Sci. Rep.* **6**, 24273; doi: 10.1038/srep24273 (2016).

## Supplementary Material

Supplementary Information

## Figures and Tables

**Table 1:** Repeated measures ANOVA (at 95% confidence intervals) results for the effects of blocks, cages, sampling dates and combination of cages and dates on the population density of cotton aphid in cotton cages field study during 2011, 2012 and 2013 at Langfang experimental station (Yellow River Region of China).

Sampling seasons									
Factor	2011			2012			2013		
	df	*F*	*P*	df	*F*	*P*	df	*F*	*P*
Block	2,10	1.37	0.299	2,10	0.34	0.719	2,10	3.72	0.062
Cage	2,10	0.60	0.552	2,10	52.14	<0.001	2,10	15.22	0.009
Date	10,50	61.36	<0.001	11,55	74.35	<0.001	11,55	62.76	<0.001
Cage × date	20,100	3.83	<0.001	22,110	1013	<0.001	22,110	7.65	<0.001

**Table 2:** Total counts of natural enemies (per 100 plants) observed in the open cotton field surveys during the growing season of 2011, 2012 and 2013 at Langfang experimental station (Yellow River Region of China).

Natural enemies	Total counts			Percentage within group (%)		
	2011	2012	2013	2011	2012	2013
Coccinellids^a^	5780	4713	4473	34.08†	20.82	26.05
Chrysopids^b^	1767	1267	513	10.42	5.60	2.99
Anthocorids^c^	2760	4267	2653	16.27	18.85	15.45
Araneae (Spiders)^d^	2813	5587	5907	16.59	24.68	34.40†
Aphid parasitoids^e^	3840	6800	3627	22.64	30.04†	21.12

^†^Dominant group of natural enemies counted in respective season.

^a^mainly *Coccinella septempunctata L., Harmonia axyridis P., Propylaea japonica T. and Adonia variegata G*.

^b^mainly *Chrysopa septempunctata W., Chrysoperla sinica T. and Chrysopa formosa B*.

^c^mainly *Orius similis Z*.

^d^mainly *Erigonidium graminicolum* S. (Linyphiidae), and Hunting spiders, *Misumenopos tricuspidata* F. (Thomisidae) and *Pardosa t-insignita* Boes. et Str. (Lycosidae).

^e^mainly *Trioxys spp*. H. and *Aphidius gifuensis A*.

**Table 3:** Seasonal mean population density of cotton aphids, predators (coccinellids, chrysopids, anthocorids, spiders) and aphid parasitoids during 2011, 2012 and 2013 at Langfang experimental station (Yellow River Region of China).

Seasonal mean population density (SEM)			
Insects guild	2011	2012	2013
Cotton aphids	1399.0 ± 76.3^c^	26345.6 ± 15948.1^a^	3430.8 ± 112.3^b^
Coccinellids	289.0 ± 15.5^a^	235.7 ± 12.4^a^	223.7 ± 100.6^b^
Chrysopids	88.3 ± 9.9^c^	63.3 ± 5.5^a^	25.7 ± 3.0^c^
Anthocorids	138.0 ± 10.8^b^	213.3 ± 12.7^a^	132.7 ± 13.1^b^
Spiders	140.7 ± 9.3^b^	279.3 ± 17.1^a^	295.3 ± 12.0^a^
Aphid parasitoids	192.0 ± 41.5^b^	340.0 ± 48.0^a^	181.3 ± 18.5^b^

Means with the same letter across sampling years are not significantly different (Student-Neuman-Keuls means separation test, α = 0.05).
