# Supplementary material for: Characterization of the natural enemy community attacking cotton aphid in the Bt cotton ecosystem in Northern China
Source: Sci Rep. 2016 Apr 14;6:24273. doi: 10.1038/srep24273 (PMC4831012; doi:10.1038/srep24273)
Supplement: Supplementary Information [file srep24273-s1.pdf]

Characterization of the natural enemy community attacking cotton aphid in the Bt cotton ecosystem in northern China

Abid Ali[1], Nicolas Desneux[2], Yanhui Lu[1], Bing Liu[1,3], Kongming Wu[1*]

[1] State Key Laboratory for Biology of Plant Diseases and Insect Pests, Institute of Plant Protection, Chinese Academy of Agricultural Sciences, Beijing 100193, P.R. China.

[2] INRA (French National Institute for Agricultural Research), Univ. Nice Sophia Antipolis, CNRS, UMR 1355-7254, Institut Sophia Agrobiotech, 06903, Sophia Antipolis, France.

[3] College of Horticulture and Plant Protection, Yangzhou University, Yangzhou, 225009, China.

*** Corresponding author:**

Kongming Wu,

Email: wukongming@caas.cn; kmwu@ippcaas.cn

Tel: +86(0)1082105551

Mailing address: State Key Laboratory for Biology of Plant Diseases and Insect Pests. Institute of Plant Protection, Chinese Academy of Agricultural Sciences, Beijing

100193, P.R. China.

Supplementary Information

Figure Legends

Figure S1: Design of surveys in field cages experimental cotton during 2011, 2012 and 2013. Design of the three treatments, i.e. natural enemy exclusion degree, in the field study; Exclusion cage: excluded all natural enemies (predators and parasitoid aphids) and fully protected aphids, Restriction cage: prevented predator movement but allowed aphid parasitoids to enter in the cages, Open field cage: allowed free access to the plants for all natural enemies. Three blocks each of 13 m x 15 m (and 2-m buffer surrounding the blocks), among 18 surveyed cages/plots per block (1-m between two plots) and each plot having 10 plants.

Figure S2: Design of surveys in open field cotton during 2011, 2012 and 2013. Three blocks each of 16 m x 15 m (and 2-m buffer surrounding the blocks), 20 surveyed fixed plots per block (1-m between two plots).

Figure S3: Comparison of seasonal mean population density (±SEM) of (**a**) cotton aphids, (**b**) coccinellids, (**c**) chrysopids, (**d**) anthocorids, (**e**) spiders and (**f**) aphid parasitoids (mummies) per 100 plants surveyed during three cotton growth stages; (i) early plant stage (May-June), (ii) middle plant stage (July), and (iii) late plant stage (August- September) from end of May to early September in the open cotton field for three growing seasons; 2011, 2012 and 2013 in Langfang experimental station (Yellow River Region of China). Black = late stage, Gray = middle stage, white = early stage. Lines are SEMs. Means with the same letter across growth stages are not significantly different (Student-Neuman-Keuls means separation test, $\alpha = 0.05$).

Figure S1:

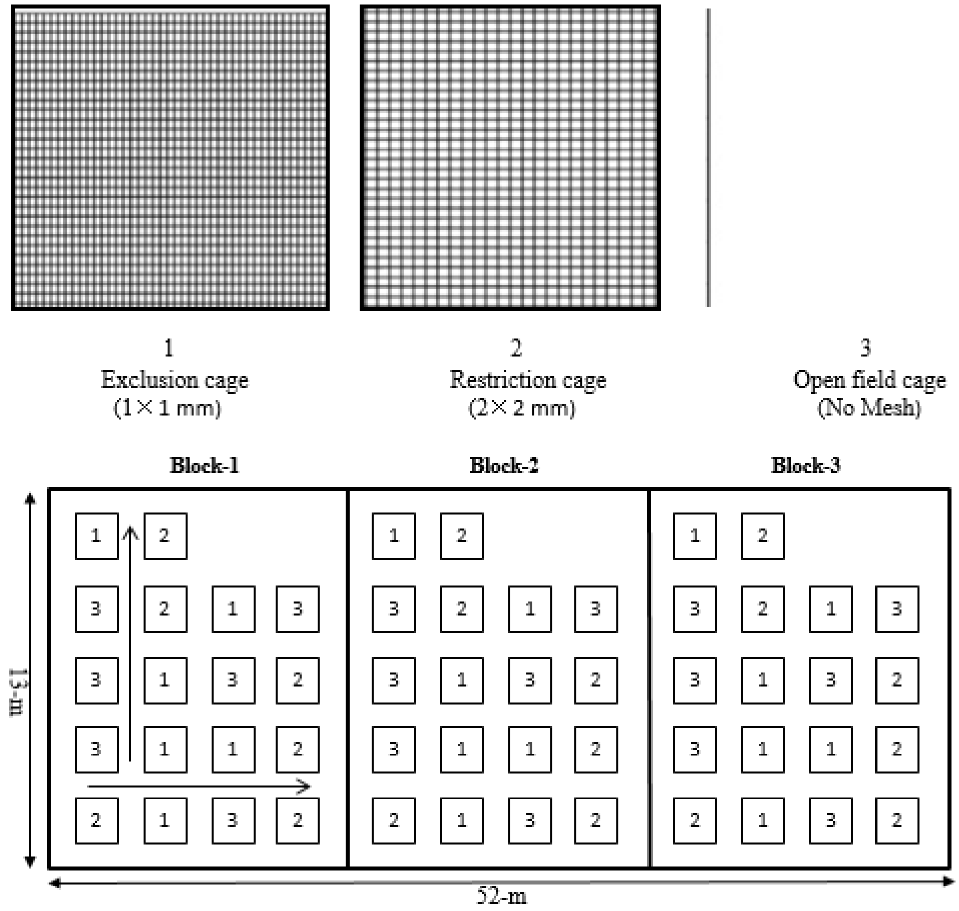
1
Exclusion cage
(1× 1 mm)
2
Restriction cage
(2× 2 mm)
3
Open field cage
(No Mesh)
Block-1
Block-2
Block-3
13-m
52-m

Figure S2:

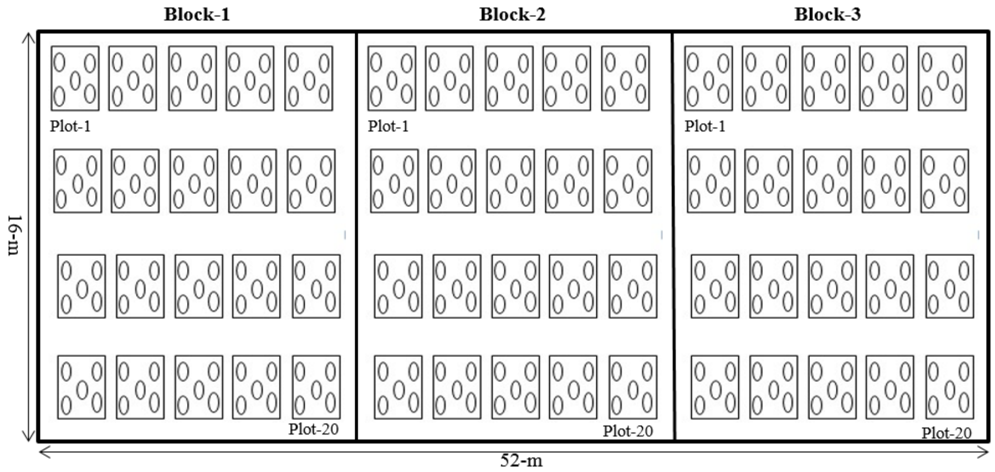
Block-1
Block-2
Block-3
Plot-1
Plot-1
Plot-1
Plot-20
Plot-20
Plot-20
16-m
52-m

Figure S3:

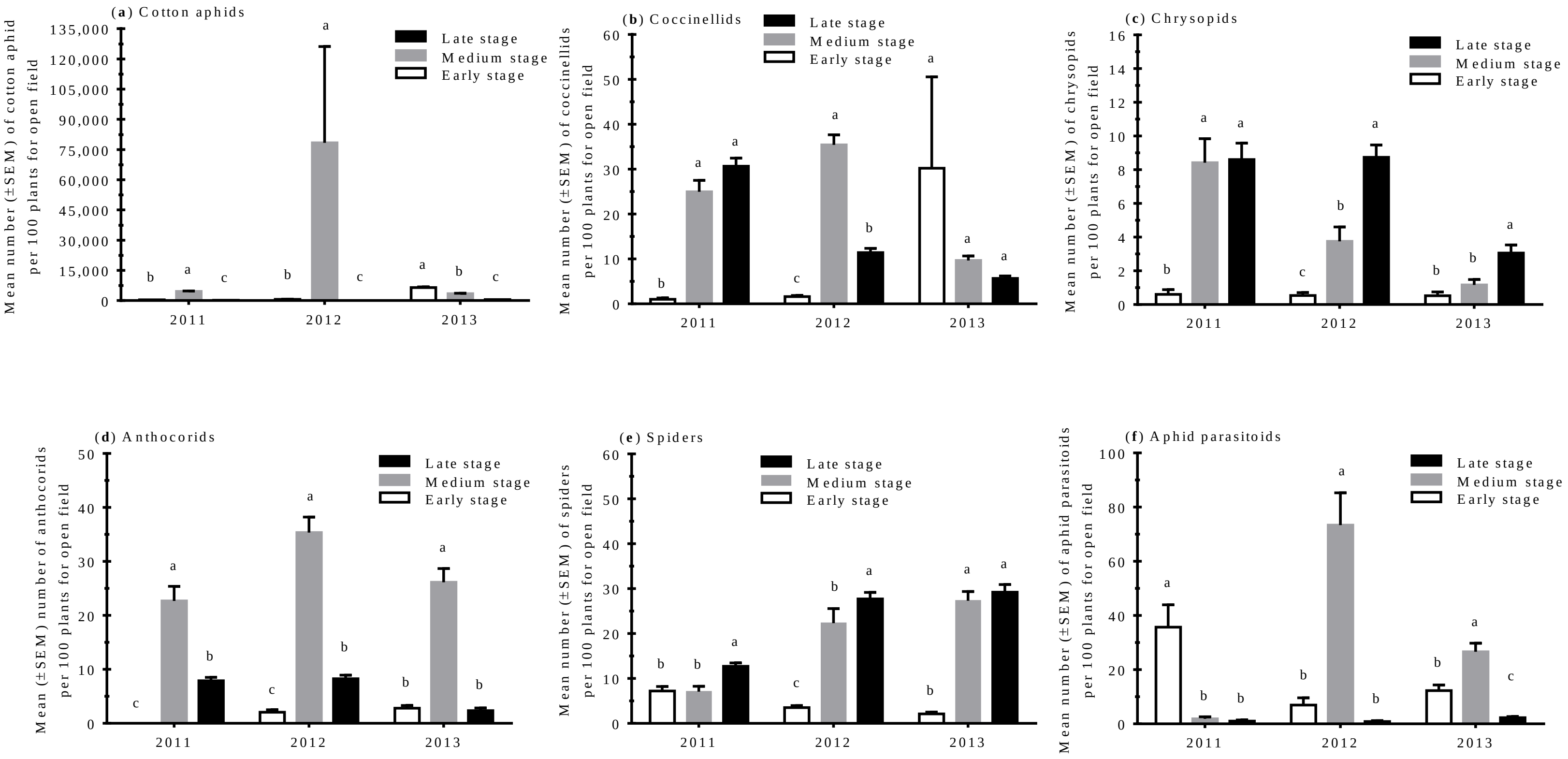
(a) Cotton aphids
Mean number (±SEM) of cotton aphid per 100 plants for open field
Late stage
Medium stage
Early stage
135,000
120,000
105,000
90,000
75,000
60,000
45,000
30,000
15,000
0
2011
2012
2013
(b) Coccinellids
Mean number (±SEM) of coccinellids per 100 plants for open field
(c) Chrysopids
Mean number (±SEM) of chrysopids per 100 plants for open field
(d) Anthocorids
Mean (±SEM) number of anthocorids per 100 plants for open field
(e) Spiders
Mean number (±SEM) of spiders per 100 plants for open field
(f) Aphid parasitoids
Mean number (±SEM) of aphid parasitoids per 100 plants for open field
